# Adaptive Sliding Mode Control Incorporating Improved Integral Compensation Mechanism for Vehicle Platoon with Input Delays

**DOI:** 10.3390/s26020615

**Published:** 2026-01-16

**Authors:** Yunpeng Ding, Yiguang Wang, Xiaojie Li

**Affiliations:** 1 Key Laboratory of Advanced Manufacturing and Automation Technology, Guilin University of Technology, Guilin 541006, China; yunpengding@glut.edu.cn (Y.D.); yiguang.wang@glut.edu.cn (Y.W.); 2 College of Mechanical and Control Engineering, Guilin University of Technology, Guilin 541006, China

**Keywords:** vehicle platoon control, input delays, integral compensation, RBFNN-based adaptive updating, unknown time-varying control coefficients

## Abstract

This study focuses on investigating the adaptive sliding mode control (SMC) problem for connected vehicles with input delays and unknown time-varying control coefficients. As a result of wear and tear of mechanical components, throttle response lags, and the internal data processing time of the controller, input delays widely exist in vehicle actuators. Since input delays may lead to instability of the vehicle platoon, an improved integral compensation mechanism (ICM) with the adjustment factor for input delays is developed to improve the platoon’s robustness. As the actuator efficiency, drive mechanism, and load of the vehicle may change during operation, the control coefficients of vehicle dynamics are usually unknown and time-varying. A novel adaptive updating mechanism utilizing a radial basis function neural network (RBFNN) is designed to deal with the unknown time-varying control coefficients, thereby improving the vehicle platoon’s tracking performance. By integrating the improved ICM and the RBFNN-based adaptive updating mechanism (RBFNN−AUM), an innovative distributed adaptive control scheme using sliding mode techniques is proposed to guarantee that the convergence of state errors to a predefined region and accomplish the vehicle platoon’s control objectives. Comparative numerical results confirm the effectiveness and superiority of the developed control strategy over existing method.

## 1. Introduction

Recently, the rapid increase in the number of automobiles has resulted in numerous traffic challenges, including traffic gridlock, energy scarcity, and environmental pollution [1,2,3,4]. Vehicle platoon control has garnered growing attention in recent years due to its potential to enhance road safety, decrease carbon emissions, optimize the utilization efficiency of resources, and increase overall traffic efficiency [5,6,7,8]. Several critical problems in the field of vehicle platoon control have been explored, for instance, string stability [9,10], spacing policy [11], communication topology [12], and nonlinear dynamics [13].

In platoon control, input delays refer to the time interval from the transmission of the control signal to the actuator’s response [14,15]. Due to wear and tear of mechanical components, throttle response lags, and the internal data processing time of the controller, input delays widely exist in vehicle actuators, potentially degrading the platoon’s tracking performance and robustness [16,17,18,19]. A number of control strategies are proposed to mitigate the adverse effects of input delays [20,21,22]. In ref. [20], a decentralized fuzzy adaptive control scheme designed for nonlinear dynamics subject to input delays is proposed to achieve ultimate boundedness of the spacing error in the neighborhood of zero. In ref. [21], a distributed backstepping control strategy is presented for hybrid uncertain dynamic system with input delays to mitigate the oscillation phenomenon caused by known input delays. In ref. [22], an adaptive neural network control method incorporating the Pade approximation technique for state-constrained system with input delays is proposed to address the constant input delays issue. Notably, the aforementioned control schemes are specifically designed for constant input delays. However, since the wear of mechanical components, throttle response lags, and controller processing delay may change during operation, input delays should be regarded as time-varying rather than constant [23,24]. Therefore, developing an innovative control approach to decrease the negative impacts of the time-varying input delays and enhance overall platoon’s performance is necessary and challenging.

As a result of the impacts from power flow rates, drive mechanisms, actuator efficiency, and vehicle loads, vehicle dynamics typically exhibit varying control coefficients, which may degrade the vehicle platoon’s tracking performance [25,26,27]. Numerous control methods have been presented to explore the coefficients of the controller in vehicle dynamics [28,29,30]. In ref. [28], a distributed backstepping control framework for control coefficients in connected environment is proposed to guarantee that each vehicle has local stability and the entire platoon possesses string stability. In ref. [29], an adaptive control method for strictly feedback nonlinear system is proposed to deal with control coefficients, thereby guaranteeing that the system state trajectories are uniformly ultimately bounded. In ref. [30], a class of enhanced Nussbaum functions for control coefficients is constructed to achieve uniform and asymptotic convergence of the output signal to zero. It is worth noting that all the above methods assume that the control coefficients are constant. However, in practice, as the actuator efficiency, drive mechanisms, power flow rates, and vehicle loads may change throughout the work process, the control coefficients may be unknown and time-varying [31,32]. Hence, designing a new adaptive control approach to effectively handle the unknown time-varying control coefficients in vehicle dynamics is both necessary and practical.

In view of the previous analysis, this work studies the platoon control problem subject to input delays and unknown time-varying control coefficients. A novel distributed adaptive sliding mode control (SMC) framework is developed to achieve the platoon’s desired performance. To decrease the negative impacts of input delays, an improved integral compensation mechanism (ICM) with the adjustment factor is designed to achieve better robustness in the vehicle platoon. A novel based on radial basis function neural network adaptive updating mechanism (RBFNN−AUM) is presented to approximate the unknown time-varying control coefficients, thereby improving the vehicle platoon’s tracking performance. The primary contributions of this study are outlined below:

1. Unlike [24], where the integral compensation gain is assumed to be one, in this work, an improved ICM with the adjustment factor for vehicle platoon is proposed to reduce the detrimental impacts of time-varying input delays and enhance the vehicle platoon’s robustness.
2. Different from [23,24] where the control coefficients are assumed to be constant, in this paper, a novel RBFNN−AUM for vehicle platoon is designed to approximate unknown time-varying control coefficients, thereby effectively enhancing the platoon’s tracking performance.
3. By incorporating the improved ICM and the RBFNN−AUM, a new distributed adaptive SMC scheme is proposed to ensure that the vehicle platoon can achieve the desired tracking performance and the ultimate control objectives.

The structure of the paper is outlined below: the dynamic model of the vehicle platoon and the control objectives is introduced in Section 2. Section 3 presents the design process of the new SMC method and the stability analysis. In Section 4, the effectiveness and superiority of the proposed control scheme is verified through numerical examples. Section 5 provides the conclusion.

In this work, the following notations are adopted: R represents the field of real numbers; exp(·) and ln(·) denote the exponential and natural logarithm functions, respectively; | · | represents the absolute value; ∥ · ∥ denotes the Euclidean norm; $C^{n}$ denotes the set of functions whose derivatives are continuous up to the *n*-th order. For the sake of presentation and without causing confusion, we will omit the dependence on parameter *t*.

## 2. Problem Formulation

**Figure 1:**
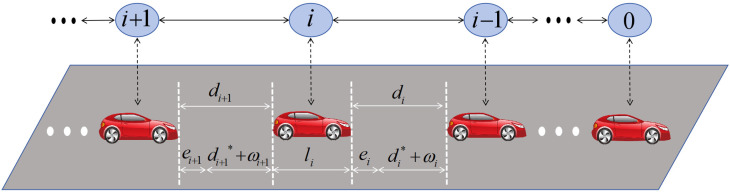
Topology structure of the platoon’s communication network.

As illustrated in Figure 1, the platoon is composed of one leader and N follower vehicles. All vehicles can obtain information from adjacent vehicles in the platoon.

### 2.1. Vehicle Dynamics

The dynamics of the leader are presented by

(1) $$\begin{array}{c} \left\{\begin{array}{cc} {\dot{x}}_{0}\left(t\right)\; & =v_{0}\left(t\right)\\ {\dot{v}}_{0}\left(t\right)\; & =a_{0}\left(t\right) \end{array}\right. \end{array}$$

where $x_{0}\left(t\right)$ is the leader’s position, $v_{0}\left(t\right)$ its velocity, and $a_{0}\left(t\right)$ its acceleration.

Considering the presence of input delays, the dynamics model of vehicle *i* are depicted by

(2) $$\begin{array}{c} \left\{\begin{array}{cc} {\dot{x}}_{i}\left(t\right) & =v_{i}\left(t\right)\\ {\dot{v}}_{i}\left(t\right) & =a_{i}\left(t\right)\\ {\dot{a}}_{i}\left(t\right) & =-\frac{1}{\kappa_{i}}a_{i}\left(t\right)-\frac{F_{mi}}{m_{i}\kappa_{i}}+\frac{\varsigma_{i}\left(t\right)}{m_{i}\kappa_{i}}u_{i}\left(t-\tau_{i}\left(t\right)\right)\\ \; & \;-\frac{m_{a}A_{i}C_{di}}{2m_{i}\kappa_{i}}v_{i}^{2}\left(t\right)-\frac{m_{a}A_{i}C_{di}}{m_{i}}v_{i}\left(t\right)a_{i}\left(t\right)\\ \; & \;+\frac{d_{mi}\left(t\right)}{m_{i}\kappa_{i}} \end{array}\right. \end{array}$$

where $x_{i}\left(t\right)$ is the *i*th vehicle’s position; $v_{i}\left(t\right)$ and $a_{i}\left(t\right)$ represent the *i*th vehicle’s velocity and acceleration, respectively; $\kappa_{i}$ refers to a constant related to time delay; $u_{i}\left(t-\tau_{i}\left(t\right)\right)$ represents the control input with time delay $\tau_{i}\left(t\right)$; $\varsigma_{i}\left(t\right)$ represents the braking input; $A_{i}$ denotes the vehicle’s cross-sectional area; $m_{i}$ is follower *i* mass; $m_{a}$ denotes the specific air mass ratio; $C_{di}$ denotes the drag constant; $F_{mi}$ is the mechanical resistance; $d_{mi}\left(t\right)$ represents the unknown external disturbance.

To facilitate controller design, (2) is reformulated as

(3) $$\left\{\begin{array}{c} {\dot{x}}_{i}\left(t\right)=v_{i}\left(t\right)\\ {\dot{v}}_{i}\left(t\right)=a_{i}\left(t\right)\\ {\dot{a}}_{i}\left(t\right)=\mu_{i}\left(t\right)u_{i}\left(t-\tau_{i}\left(t\right)\right)+f_{i}\left(v_{i}\left(t\right),a_{i}\left(t\right)\right)+\varpi_{i}\left(t\right) \end{array}\right.$$

where $\mu_{i}\left(t\right)=$$\varsigma_{i}\left(t\right)/\left(m_{i}\kappa_{i}\right)$ represents the unknown time-varying control coefficient; $\varpi_{i}\left(t\right)$=${dm}_{i}\left(t\right)$ $/(m_{i}\kappa_{i})$ represents the reformulated external disturbance; $f_{i}(v_{i}\left(t\right),$$a_{i}\left(t\right))=-$$a_{i}\left(t\right)$$/\kappa_{i}$-$F_{mi}/\left(m_{i}\kappa_{i}\right)$-$A_{i}C_{di}v_{i}^{2}\left(t\right)/\left(2m_{i}\kappa_{i}\right)$-$m_{a}A_{i}C_{di}v_{i}\left(t\right)a_{i}\left(t\right)/$$m_{i}$ represents the unknown nonlinear function of the *i*th vehicle. For the sake of generality, one can assume that

(4) $${\bar{\mu }}_{i}\geq \mu_{i}\left(t\right)\geq {\underline{\mu }}_{i}>0$$

where ${\bar{\mu }}_{i}$ the upper bounds of $\mu_{i}\left(t\right)$; ${\underline{\mu }}_{i}$ represents lower bounds of $\mu_{i}\left(t\right)$.

### 2.2. RBFNN Approximation

A RBFNN is utilized to approximate $\mu_{i}\left(t\right)$ and $f_{i}$($v_{i}\left(t\right)$, $a_{i}\left(t\right)$) in this section. According to the technique mentioned in [33], it is possible to approximate $\mu_{i}\left(t\right)$ and $f_{i}$($v_{i}\left(t\right)$, $a_{i}\left(t\right)$) as

(5) $$\left\{\begin{array}{c} {\hat{\mu }}_{i}\left(t\right)={\hat{W}}_{\mu_{i}}^{T}\phi_{\mu_{i}}\left(Z_{i}\right)+\varepsilon_{\mu_{i}}\\ {\hat{f}}_{i}\left(v_{i}\left(t\right),a_{i}\left(t\right)\right)={\hat{W}}_{f_{i}}^{T}\phi_{f_{i}}\left(Z_{i}\right)+\varepsilon_{f_{i}} \end{array}\right.$$

where ${\hat{W}}_{f_{i}}\in R^{q_{i}}$ and ${\hat{W}}_{\mu_{i}}\in R^{q_{i}}$ denote the weight vectors with $q_{i}$ being the number of neurons; $Z_{i}={\left[z_{1},z_{2},\ldots ,z_{n}\right]}^{\;T}$ is the input vector; $\varepsilon_{\mu_{i}}$ and $\varepsilon_{f_{i}}$ denote the approximation errors that satisfy $|\varepsilon_{\mu_{i}}\left|\;\leq \;\right|\varepsilon_{max}|$ and $|\varepsilon_{f_{i}}\left|\;\leq \;\right|\varepsilon_{max}|$, respectively, where $\varepsilon_{max}$ is an unknown bound parameter.

Generally, RBFNN can smoothly approximate any continuous function f(Z) on a compact set $Z\in R^{q}$ to arbitrary accuracy as [34]

(6) $$f\left(Z\right)=W^{T}\phi \left(Z\right)+\varepsilon ,\;\forall Z\in {Ω}_{Z}\subset R^{q}$$

where *W* is the optimal weight vector and ε is the smallest approximation error. The Gaussian basis function vector $\phi_{ik}\left(Z_{i}\right)$ is

(7) $$\phi_{ik}\left(Z_{i}\right)=\exp\left[-\frac{{\left(Z_{i}-c_{k}\right)}^{T}\left(Z_{i}-c_{k}\right)}{2{b_{k}}^{2}}\right]$$

where $b_{k}$ is the width value; $c_{k}={\left[c_{k1},c_{k2},\ldots ,c_{kn}\right]}^{\;T}$ represents the center of receptive field; $k\in \{1,2,\ldots ,q_{i}\}$.

The optimal weight value of RBFNN is given by [34]

(8) $$W^{*}=\arg\min_{\begin{array}{c} \hat{W}\in {Ω}_{f} \end{array}}\left[\underset{\begin{array}{c} Z\in \phi_{Z} \end{array}}{\sup}\left|\hat{f}\left(Z|\hat{W}\right)-f\left(Z\right)\right|\right]$$

where ${Ω}_{f}=\{\hat{W}\;:\;∥\hat{W}∥\;\leq M\}$ is the valid domain of the parameter; *M* is a design parameter; and $\phi_{Z}\subset R^{n}$ is an allowable set of the state vector.

With the optimal weight value, one has

(9) $$|f\left(Z\right)-W^{T}\phi \left(Z\right)|=|\varepsilon |\;\leq \;|\varepsilon_{max}|$$

**Remark** **1** ([35])**.** By the Weierstrass Approximation Theorem, for functions defined on a compact domain Ω, there exists a sufficiently large constant $q_{i}^{*}>0$ such that for any $q_{i}>q_{i}^{*}$, it is always possible to find ideal $W_{f_{i}},W_{\mu_{i}},\phi_{f_{i}}\left(\cdot \right)$, and $\phi_{\mu_{i}}\left(\cdot \right)$, such that the maximum approximation errors $\max_{v_{i},a_{i}\in Ω}\left|\varepsilon_{fi}\right|$ and $\max_{v_{i},a_{i}\in Ω}\left|\varepsilon_{\mu_{i}}\right|$ in *(5)* are small enough.

### 2.3. Control Objective

A novel adaptive SMC scheme for vehicle platoon subject to input delays and unknown time-varying control coefficients is proposed, aiming to achieve the objectives listed below.

1. Individual stability [36]: Each vehicle maintains the desired inter-vehicle distance while achieving consensus on velocity with the leader.
2. String stability [37]: The vehicle platoon achieves string stability with respect to the spacing error $e_{i}\left(t\right)$, when
  (10) $$\left|\frac{E_{i+1}\left(s\right)}{E_{i}\left(s\right)}\right|\leq 1,\;i=1,\ldots ,N-1$$
  where $E_{i}\left(s\right)$ is the Laplace transform of $e_{i}\left(t\right)$, *s* is the Laplace operator.
3. Prescribed tracking performance [38]: $e_{i}\left(t\right)$ converges to a given region in predefined time $T_{i}>0$, that is,
  (11) $$-\ell_{\min}\rho \left(t\right)<e_{i}\left(t\right)<\ell_{\max}\rho \left(t\right)\;,\;\forall \;t\geq T_{i}$$
  where ρ(t)>0 is a performance function, $\ell_{\min}$ and $\ell_{\max}$ are design parameters.

To achieve the aforementioned objectives, some assumptions, lemma and definition need to be given as follows.

**Assumption** **1.** *$\varpi_{i\max}>0$ is an unknown constant, and satisfies $|\varpi_{i}\left(t\right)|\leq \varpi_{i\max}$.*

**Assumption** **2.** *Assume that $\tau_{i}\left(t\right)$ satisfies $|\tau_{i}\left(t\right)|\leq \tau_{i\max}$ for a known positive constant $\tau_{i\max}$.*

**Lemma** **1** ([39])**.** *Given any g∈R, there exists a constant ι>0 that satisfies*

(12) $$0\leq \left|g\right|-g\;\tanh\;\left(\frac{g}{\iota }\right)\leq 0.2785\iota$$

**Definition** **1** ([40])**.** *$r_{i}\left(t\right)$ represents a shift function, which meets the conditions listed below.*

1. *When $t\in [0,T_{i})$, the function $r_{i}\left(t\right)$ is monotonically increasing; When $t\in [T_{i},+\infty )$, $r_{i}\left(t\right)=1$, with $T_{i}>0$ denotes the settling time.*
2. *For j=0,…,n+1, $r_{i}^{\left(j\right)}\left(t\right)\in C^{n+1-j}$, and is bounded for all t∈[0,+∞).*

## 3. Controller Design and Stability Analysis

### 3.1. Spacing Policy

As depicted in Figure 1, the inter-vehicle spacing $d_{i}\left(t\right)$ is formulated as

(13) $$d_{i}\left(t\right)=x_{i-1}\left(t\right)-x_{i}\left(t\right)-y_{i}\;,\;i=1,\ldots ,N$$

where $y_{i}$ refers to the length of the *i*th vehicle. To mitigate the impact of input delays, an improved constant time headway spacing $d_{i}^{*}\left(t\right)$ is formulated as

(14) $${d_{i}}^{*}\left(t\right)=\Delta_{i}+hv_{i}\left(t\right)+K_{i}\int_{0}^{t}\beta_{i}\left(t\right)d\tau$$

where $\Delta_{i}$ represents the standstill distance; $0\leq K_{i}\leq 1$ is the adjustment factor for delay compensation; *h* is the headway time, and $\beta_{i}\left(t\right)$ is the delay compensation variable updated by

(15) $${\dot{\beta }}_{i}\left(t\right)=-h\mu_{i}\left(t\right)\left(u_{i}\left(t-\tau_{i\max}\right)-u_{i}\left(t\right)\right)-c_{i}\beta_{i}\left(t\right)$$

where $c_{i}$ is a positive constant; $u_{i}\left(t-\tau_{i\max}\right)$ represent the control input with the maximum delay.

Then, the spacing error $e_{i}\left(t\right)$ is given by

(16) $$e_{i}\left(t\right)=d_{i}\left(t\right)-{d_{i}}^{*}\left(t\right)$$

**Remark** **2.** *$K_{i}\in \left[0,1\right]$ can be considered as an indicator of the degree of compensation for input delays. When $K_{i}$ approaches zero, the input delays compensation has almost no feedback, and $K_{i}=1$ indicates the maximum compensation for input delays.*

### 3.2. Controller Design

Under realistic operating conditions, the initial conditions of the spacing error may differ and cannot be precisely measured. To deal with the conditions of unknown initial spacing error, an auxiliary variable $w_{i}$ is employed as

(17) $$e_{i}^{m}\left(t\right)=e_{i}\left(t\right)-w_{i}=d_{i}\left(t\right)-{d_{i}}^{*}\left(t\right)-w_{i}$$

where $w_{i}=t\left(1-r_{i}\left(t\right)\right){\dot{e}}_{i}\left(0\right)+\left(1-r_{i}\left(t\right)+t{\dot{r}}_{i}\left(t\right)\right)e_{i}\left(0\right)$, $e_{i}^{m}\left(t\right)$ denotes the modified spacing error. According to Definition 1, $r_{i}\left(t\right)$ is designed as

(18) $$r_{i}\left(t\right)=\left\{\begin{array}{c} 1-{\left(\frac{T_{i}-t}{T_{i}}\right)}^{\Gamma }\;,\;t\in \left[0,T_{i}\right)\\ 1\;,\;t\in [T_{i},+\infty ) \end{array}\right.$$

where Γ is constant.

Combining (17) and (18), we can obtain

(19) $$e_{i}^{m}\left(0\right)=0\;,\;{\dot{e}}_{i}^{m}\left(0\right)=0$$

which can make the modified spacing error $e_{i}^{m}\left(t\right)$ converges to the prescribed region under any initial spacing error conditions.

From (19), we can see that (11) can be converted to $-\ell_{\min}\rho \left(t\right)<e_{i}^{m}\left(t\right)<\ell_{\max}\rho \left(t\right)$ for all t∈[0,+∞). Since $e_{i}^{m}\left(t\right)$ is restricted and cannot be directly employed for controller design, an error mapping transformation is adopted as [41]

(20) $$e_{i}^{m}\left(t\right)=\Pi_{i}\left(\psi_{i}\right)\rho$$

where $\psi_{i}$ is the unconstrained transformation error, $\Pi_{i}\left(\psi_{i}\right)$ is a smooth monotonically increasing function described as

(21) $$\Pi_{i}\left(\psi_{i}\right)=\frac{\ell_{\max}\exp\left(\psi_{i}\right)-\ell_{\min}\exp\left(-\psi_{i}\right)}{\exp\left(\psi_{i}\right)+\exp\left(-\psi_{i}\right)}$$

**Remark** **3.** *The function $\Pi_{i}\left(\psi_{i}\right)$ defined in *(21)* is smooth, continuous, and strictly monotonically increasing for $\psi_{i}\in R$. Its derivative is given by*

(22) $$\frac{d}{d\psi_{i}}\Pi_{i}\left(\psi_{i}\right)=\frac{2(\ell_{\max}+\ell_{\min})}{{\left[\exp\left(\psi_{i}\right)+\exp\left(-\psi_{i}\right)\right]}^{2}}>0,\;\forall \psi_{i}\in R,$$

*The mapping function $\Pi_{i}\left(\psi_{i}\right)$ is defined for all $\psi_{i}\in R$. By analyzing its derivative, it can be shown that $\Pi_{i}\left(\psi_{i}\right)$ is strictly increasing on R. Moreover, when $\psi_{i}\to -\infty$, $\Pi_{i}\left(\psi_{i}\right)\to -\ell_{\min}$, and when $\psi_{i}\to +\infty$, $\Pi_{i}\left(\psi_{i}\right)\to \ell_{\max}$. Since $\Pi_{i}\left(\psi_{i}\right)$ is continuous and strictly increasing, it constitutes a bijective mapping from R onto $\left(-\ell_{\min},\ell_{\max}\right)$. Given that ρ(t)>0 for all t, the transformation $e_{i}^{m}\left(t\right)$ also remains bijective, thereby ensuring the rigor and rationality of this mapping.*

Based on (20) and (21), we have

(23) $$\psi_{i}=\frac{1}{2}\ln\left(\frac{\Pi_{i}+\ell_{\min}}{\ell_{\max}-\Pi_{i}}\right)$$

Using $\psi_{i}$, the sliding mode surface is given by

(24) $$s_{i}={\dot{\psi }}_{i}+\lambda \psi_{i}$$

where λ is the Hurwitz constant. A coupling sliding mode surface is constructed to guarantee string stability, defined as

(25) $$\Psi_{i}=\left\{\begin{array}{cc} \delta s_{i}-s_{i+1}, & i=1,\ldots ,N-1\\ \delta s_{i}, & i=N \end{array}\right.$$

with the coupling coefficient δ satisfies 0<δ≤1.

According to (20) and (23), one has

(26) $$\begin{array}{cc} {\dot{\psi }}_{i} & =\frac{1}{2}\frac{1}{\Pi_{i}+\ell_{\min}}\frac{1}{\rho }\left({\dot{e}}_{i}^{m}-\frac{e_{i}^{m}\dot{\rho }}{\rho }\right)+\frac{1}{2}\frac{1}{\ell_{\max}-\Pi_{i}}\frac{1}{\rho }\left({\dot{e}}_{i}^{m}-\frac{e_{i}^{m}\dot{\rho }}{\rho }\right)\\ \; & =\frac{1}{2\rho }\left(\frac{\ell_{\min}+\ell_{\max}}{\left(\Pi_{i}+\ell_{\min}\right)\left(\ell_{\max}-\Pi_{i}\right)}\right)\left({\dot{e}}_{i}^{m}-\frac{e_{i}^{m}\dot{\rho }}{\rho }\right)\\ \; & =\varphi \left({\dot{e}}_{i}^{m}-\frac{e_{i}^{m}\dot{\rho }}{\rho }\right) \end{array}$$

where $\varphi_{i}=\frac{1}{2\rho }\left(\frac{\ell_{\min}+\ell_{\max}}{\left(\Pi_{i}+\ell_{\min}\right)\left(\ell_{\max}-\Pi_{i}\right)}\right).$

Then

(27) $$\begin{array}{cc} {\ddot{\psi }}_{i} & ={\dot{\varphi }}_{i}{\dot{e}}_{i}^{m}-\frac{{\dot{\varphi }}_{i}e_{i}^{m}\dot{\rho }}{\rho }+\varphi_{i}\left({\ddot{e}}_{i}^{m}-\frac{\left({\dot{e}}_{i}^{m}\dot{\rho }+e_{i}^{m}\ddot{\rho }\right)\rho -e_{i}^{m}{\dot{\rho }}^{2}}{\rho^{2}}\right)\\ \; & =\varphi_{i}\left({\ddot{d}}_{i}-{\ddot{d}}_{i}^{*}-{\ddot{w}}_{i}-\frac{\left({\dot{e}}_{i}^{m}\dot{\rho }+e_{i}^{m}\ddot{\rho }\right)\rho -e_{i}^{m}{\dot{\rho }}^{2}}{\rho^{2}}\right)+{\dot{\varphi }}_{i}{\dot{e}}_{i}^{m}-\frac{{\dot{\varphi }}_{i}e_{i}^{m}\dot{\rho }}{\rho } \end{array}$$

Thus, for i=1,…,N−1, differentiating $\Psi_{i}$

(28) $$\begin{array}{cc} {\dot{\Psi }}_{i} & =\delta \left(\varphi_{i}K_{i}c_{i}\beta_{i}-\varphi_{i}\frac{\left({\dot{e}}_{i}^{m}\dot{\rho }+e_{i}^{m}\ddot{\rho }\right)\rho -e_{i}^{m}{\dot{\rho }}^{2}}{\rho^{2}}\right)\\ \; & \;+\delta \left({\dot{\varphi }}_{i}{\dot{e}}_{i}^{m}-\frac{{\dot{\varphi }}_{i}e_{i}^{m}\dot{\rho }}{\rho }+\lambda {\dot{\psi }}_{i}\right)-{\dot{s}}_{i+1}\\ \; & \;+\delta \varphi_{i}\left(a_{i-1}-a_{i}-h\left(K_{i}\mu_{i}u_{i}\left(t\right)+f_{i}+\varpi_{i}\right)\right)\\ \; & \;-\delta \varphi_{i}\left({\ddot{w}}_{i}+h\mu_{i}u_{i}\left(t-\tau_{i}\right)-K_{i}h\mu_{i}u_{i}\left(t-\tau_{i\max}\right)\right) \end{array}$$

For i=N, ${\dot{\Psi }}_{i}$ is

(29) $$\begin{array}{cc} {\dot{\Psi }}_{i} & =\delta \left(\varphi_{i}K_{i}c_{i}\beta_{i}-\varphi_{i}\frac{\left({\dot{e}}_{i}^{m}\dot{\rho }+e_{i}^{m}\ddot{\rho }\right)\rho -e_{i}^{m}{\dot{\rho }}^{2}}{\rho^{2}}\right)\\ \; & \;+\delta \left({\dot{\varphi }}_{i}{\dot{e}}_{i}^{m}-\frac{{\dot{\varphi }}_{i}e_{i}^{m}\dot{\rho }}{\rho }+\lambda {\dot{\psi }}_{i}\right)\\ \; & \;+\delta \varphi_{i}\left(a_{i-1}-a_{i}-h\left(K_{i}\mu_{i}u_{i}\left(t\right)+f_{i}+\varpi_{i}\right)\right)\\ \; & \;-\delta \varphi_{i}\left({\ddot{w}}_{i}+h\mu_{i}u_{i}\left(t-\tau_{i}\right)-K_{i}h\mu_{i}u_{i}\left(t-\tau_{i\max}\right)\right) \end{array}$$

According to (5), we can obtain

(30) $${\dot{\Psi }}_{i}=\delta \left(-\varphi_{i}h\left(K_{i}\mu_{i}u_{i}\left(t\right)+W_{f_{i}}^{T}\phi_{f_{i}}+Y_{i}\right)+R_{i}\right)$$

with

(31) $$R_{i}=\left\{\begin{array}{c} \varphi_{i}K_{i}c_{i}\beta_{i}-\varphi_{i}\frac{\left({\dot{e}}_{i}^{m}\dot{\rho }+e_{i}^{m}\ddot{\rho }\right)\rho -e_{i}^{m}{\dot{\rho }}^{2}}{\rho^{2}}+{\dot{\varphi }}_{i}\left({\dot{e}}_{i}^{m}-\frac{{\dot{\rho }}_{i}e_{i}^{m}}{\rho }\right)+\varphi_{i}\left(a_{i-1}-a_{i}-{\ddot{w}}_{i}\right)\\ \;-\frac{{\dot{s}}_{i+1}}{\delta }+\lambda {\dot{\psi }}_{i},\;i=1,\ldots ,N-1\\ \varphi_{i}K_{i}c_{i}\beta_{i}-\varphi_{i}\frac{\left({\dot{e}}_{i}^{m}\dot{\rho }+e_{i}^{m}\ddot{\rho }\right)\rho -e_{i}^{m}{\dot{\rho }}^{2}}{\rho^{2}}+{\dot{\varphi }}_{i}\left({\dot{e}}_{i}^{m}-\frac{\dot{\rho }e_{i}^{m}}{\rho }\right)+\varphi_{i}\left(a_{i-1}-a_{i}-{\ddot{w}}_{i}\right)\\ \;+\lambda {\dot{\psi }}_{i},\;i=N \end{array}\right.$$

(32) $$\begin{array}{c} Y_{i}=\;\varepsilon_{f}+\varpi_{i}+h\mu_{i}u_{i}\left(t-\tau_{i}\right)-K_{i}h\mu_{i}u_{i}\left(t-\tau_{i\max}\right) \end{array}$$

where $Y_{i}$ is completely unknown, $R_{i}$ is available for control input design.

The control input $u_{i}\left(t\right)$ is constructed as

(33) $$\begin{array}{cc} u_{i}\left(t\right)= & \frac{1}{K_{i}{\hat{\mu }}_{i}}\left(\frac{k_{i}\Psi_{i}}{\delta h\varphi_{i}}+\frac{R_{i}}{h\varphi_{i}}+\frac{\delta h\varphi_{i}\Psi_{i}}{4\gamma_{i}}{\hat{\theta }}_{i}\phi_{fi}^{T}\phi_{fi}+{\hat{\psi }}_{i}\tanh\left(\frac{\Psi_{i}\varphi_{i}}{\zeta_{i}}\right)\right) \end{array}$$

where ${\hat{\mu }}_{i}$, ${\hat{\theta }}_{i}$, and ${\hat{\psi }}_{i}$ are the estimated values of $\mu_{i}$, $\theta_{i}$, and $\psi_{i}$, respectively; $\theta_{i}={∥W_{f_{i}}∥}^{2}=W_{f_{i}}^{T}W_{f_{i}}$; $k_{i}>0$, $\gamma_{i}>0$, and $\zeta_{i}>0$ are all constants;

The updating laws for ${\hat{\theta }}_{i}$, ${\hat{\psi }}_{i}$, and ${\hat{W}}_{\mu_{i}}$ are designed as

(34) $${\dot{\hat{\theta }}}_{i}=F_{\theta_{i}}\left(\frac{1}{4\gamma_{i}}\delta^{2}h^{2}{\varphi_{i}}^{2}{\Psi_{i}}^{2}\phi_{fi}^{T}\phi_{fi}-\sigma_{\theta_{i}}{\hat{\theta }}_{i}\right)$$

(35) $${\dot{\hat{\psi }}}_{i}=F_{\psi_{i}}\left(\delta h\varphi_{i}\Psi_{i}\tanh\left(\frac{\Psi_{i}\varphi_{i}}{\zeta_{i}}\right)-\sigma_{\psi_{i}}{\hat{\psi }}_{i}\right)$$

(36) $${\dot{\hat{W}}}_{\mu_{i}}=\left\{\begin{array}{cc} \; & -F_{\mu_{i}}\left(K_{i}\delta h\varphi_{i}\Psi_{i}\phi_{\mu_{i}}u_{i}+\sigma_{\mu_{i}}{\hat{W}}_{\mu_{i}}\right),\mathrm{if}\;{\hat{\mu }}_{i}>{\underline{\mu }}_{i}\\ \; & -F_{\mu_{i}}\left(K_{i}\delta h\varphi_{i}\Psi_{i}\phi_{\mu_{i}}u_{i}+\sigma_{\mu_{i}}{\hat{W}}_{\mu_{i}}\right),\mathrm{if}\;{\hat{\mu }}_{i}={\underline{\mu }}_{i}\;\mathrm{and}\;\left(K_{i}\delta h\varphi_{i}\Psi_{i}\phi_{\mu_{i}}u_{i}+\sigma_{\mu_{i}}{\hat{W}}_{\mu_{i}}\right)<0\\ \; & 0,\;\mathrm{if}\;{\hat{\mu }}_{i}={\underline{\mu }}_{i}\;\mathrm{and}\;\left(K_{i}\delta h\varphi_{i}\Psi_{i}\phi_{\mu_{i}}u_{i}+\sigma_{\mu_{i}}{\hat{W}}_{\mu_{i}}\right)\geq 0 \end{array}\right.$$

where $F_{\theta_{i}}>0$, $F_{\psi_{i}}>0$, $F_{\mu_{i}}>0$, $\sigma_{\theta_{i}}>0$, and $\sigma_{\psi_{i}}>0$ are design parameters; ${\underline{\mu }}_{i}>0$ is the minimum value of $\mu_{i}$, and $\sigma_{\mu_{i}}>0$.

**Remark** **4.** *The updating law *(36)* employs a projection operator to ensure the estimated control coefficient ${\hat{\mu }}_{i}\left(t\right)={\hat{W}}_{\mu i}^{T}\phi_{\mu i}$ satisfies ${\hat{\mu }}_{i}\left(t\right)\geq \underline{\mu }>0$ for all t≥0. Specifically, when ${\hat{\mu }}_{i}$ reaches its known physical lower bound $\underline{\mu }$ or the updating law would drive it further below this bound, the parameter updating is suspended. This mechanism is critical for preventing singularity in the control law (33).*

### 3.3. Stability Analysis

**Theorem** **1.** *For vehicle platoon *(3)* with input delays and unknown time-varying control coefficients, the developed controller *(33)* and updating mechanisms *(34)–(36)* can ensure that $e_{i}^{m}$ converges to a prescribed region in predefined time and the individual stability. Moreover, when 0<δ≤1 is satisfied, string stability can also be achieved.*

#### 3.3.1. Individual Stability

A Lyapunov function is considered to be

(37) $$V=\frac{1}{2}\sum_{i=1}^{N}\left(\Psi_{i}^{2}+F_{\theta_{i}}^{-1}{\tilde{\theta }}_{i}^{2}+F_{\psi_{i}}^{-1}{\tilde{\psi }}_{i}^{2}+F_{\mu_{i}}^{-1}{{\tilde{W}}_{\mu_{i}}}^{T}{\tilde{W}}_{\mu_{i}}+\beta_{i}^{2}\right)$$

where ${\tilde{\theta }}_{i}=\theta_{i}-{\hat{\theta }}_{i}$, ${\tilde{\psi }}_{i}=\psi_{i}-\hat{\psi_{i}}$ and ${\tilde{W}}_{\mu_{i}}=W_{\mu_{i}}-{\hat{W}}_{\mu_{i}}$ are the estimation errors.

According to (15) and (30), we have

(38) $$\begin{array}{cc} \dot{V}= & \sum_{i=1}^{N}\left(\Psi_{i}{\dot{\Psi }}_{i}-F_{\theta_{i}}^{-1}{\tilde{\theta }}_{i}{\dot{\hat{\theta }}}_{i}-F_{\psi_{i}}^{-1}{\tilde{\psi }}_{i}{\dot{\hat{\psi }}}_{i}-F_{\mu_{i}}^{-1}{\tilde{W}}_{\mu_{i}}^{T}{\dot{\hat{W}}}_{\mu_{i}}+\beta_{i}{\dot{\beta }}_{i}\right)\\ = & -\sum_{i=1}^{N}(\delta h\varphi_{i}\Psi_{i}W_{f_{i}}^{T}\phi_{f_{i}}+K_{i}\delta h\varphi_{i}\Psi_{i}{\tilde{\mu }}_{i}u_{i}+\frac{\delta^{2}h^{2}\varphi_{i}^{2}\Psi_{i}^{2}{\hat{\theta }}_{i}\phi_{f_{i}}^{T}\phi_{f_{i}}}{4\gamma_{i}}\\ \; & \;\;+\delta h\varphi_{i}\Psi_{i}Y_{i}+k_{i}\Psi_{i}^{2}+\delta h\varphi_{i}\Psi_{i}\hat{\psi }\tanh\left(\frac{\Psi_{i}\varphi_{i}}{\zeta_{i}}\right))\\ \; & \;\;-\sum_{i=1}^{N}F_{\theta_{i}}^{-1}{\tilde{\theta }}_{i}{\dot{\hat{\theta }}}_{i}-\sum_{i=1}^{N}F_{\psi_{i}}^{-1}{\tilde{\psi }}_{i}{\dot{\hat{\psi }}}_{i}-\sum_{i=1}^{N}F_{\mu_{i}}^{-1}{\tilde{W}}_{\mu_{i}}^{T}{\dot{\hat{W}}}_{\mu_{i}}\\ \; & \;\;+\sum_{i=1}^{N}\left(-c_{i}\beta_{i}^{2}-h\beta_{i}\mu_{i}\left(u_{i}\left(t-\tau_{i\max}\right)-u_{i}\left(t\right)\right)\right) \end{array}$$

Using the updating mechanisms (34)–(36), we can obtain

(39) $$\begin{array}{cc} \dot{V}= & -\sum_{i=1}^{N}(k_{i}\Psi_{i}^{2}+\frac{{\hat{\theta }}_{i}}{4\gamma_{i}}\delta^{2}\varphi_{i}^{2}h^{2}\Psi_{i}^{2}\phi_{f_{i}}^{T}\phi_{f_{i}}+\delta h\varphi_{i}\Psi_{i}Y_{i}+\delta h\varphi_{i}\Psi_{i}{\hat{\psi }}_{i}\tanh\left(\frac{\Psi_{i}\varphi_{i}}{\zeta_{i}}\right)\\ \; & \;\;+\delta h\varphi_{i}\Psi_{i}W_{f_{i}}^{T}\phi_{f_{i}})+\sum_{i=1}^{N}\left(-c_{i}\beta_{i}^{2}-h\beta_{i}\mu_{i}\left(u_{i}\left(t-\tau_{i\max}\right)-u_{i}\left(t\right)\right)\right)\\ \; & \;\;-\sum_{i=1}^{N}\left(\frac{{\tilde{\theta }}_{i}}{4\gamma_{i}}\delta^{2}h^{2}\varphi_{i}^{2}\Psi_{i}^{2}\phi_{f_{i}}^{T}\phi_{f_{i}}-\sigma_{\theta_{i}}{\tilde{\theta }}_{i}{\hat{\theta }}_{i}\right)\\ \; & \;\;-\sum_{i=1}^{N}\left({\tilde{\psi }}_{i}\delta h\varphi_{i}\Psi_{i}\tanh\left(\frac{\Psi_{i}\varphi_{i}}{\zeta_{i}}\right)-\sigma_{\psi_{i}}{\tilde{\psi }}_{i}{\hat{\psi }}_{i}\right)\\ \; & \;\;+\sum_{i=1}^{N}\left\{\begin{array}{c} \sigma_{\mu_{i}}{\tilde{W}}_{\mu_{i}}^{T}{\hat{W}}_{\mu_{i}},\;\;\mathrm{if}\;{\hat{\mu }}_{i}>{\underline{\mu }}_{i},\\ \sigma_{\mu_{i}}{\tilde{W}}_{\mu_{i}}^{T}{\hat{W}}_{\mu_{i}},\;\;\mathrm{if}\;{\hat{\mu }}_{i}={\underline{\mu }}_{i}\;\mathrm{and}\;\left(K_{i}\delta h\varphi_{i}\Psi_{i}\phi_{\mu_{i}}u_{i}+\sigma_{\mu_{i}}{\hat{W}}_{\mu_{i}}\right)<0,\\ -K_{i}\delta h\varphi_{i}\Psi_{i}{\tilde{W}}_{\mu_{i}}^{T}\phi_{\mu_{i}}u_{i},\\ \;\;\mathrm{if}\;{\hat{\mu }}_{i}={\underline{\mu }}_{i}\;\mathrm{and}\;\left(K_{i}\delta h\varphi_{i}\Psi_{i}\phi_{\mu_{i}}u_{i}+\sigma_{\mu_{i}}{\hat{W}}_{\mu_{i}}\right)\geq 0 \end{array}\right. \end{array}$$

Considering $\sigma_{\mu_{i}}$${\hat{W}}_{\mu_{i}}$ ≥$\;-K_{i}\delta h\varphi_{i}\Psi_{i}\phi_{\mu_{i}}u_{i}$, if $(K_{i}\delta h\varphi_{i}$$\Psi_{i}\phi_{\mu_{i}}u_{i}+\sigma_{\mu_{i}}{\hat{W}}_{\mu_{i}})\geq 0$, we have

(40) $$\begin{array}{cc} \sigma_{\mu_{i}}{\tilde{W}}_{\mu_{i}}^{T}{\hat{W}}_{\mu_{i}} & \geq -K_{i}\delta h\varphi_{i}\Psi_{i}{\tilde{W}}_{\mu_{i}}^{T}\phi_{\mu_{i}}u_{i},\\ \; & \;\;\mathrm{if}\;{\hat{\mu }}_{i}={\underline{\mu }}_{i}\;\mathrm{and}\;\left(K_{i}\delta h\varphi_{i}\Psi_{i}\phi_{\mu_{i}}u_{i}+\sigma_{\mu_{i}}{\hat{W}}_{\mu_{i}}\right)\geq 0 \end{array}$$

Then, (39) can be described as

(41) $$\begin{array}{cc} \dot{V}\leq & \sum_{i=1}^{N}\left(-k_{i}\Psi_{i}^{2}+\sigma_{\theta_{i}}{\tilde{\theta }}_{i}{\hat{\theta }}_{i}+\sigma_{\psi_{i}}{\tilde{\psi }}_{i}{\hat{\psi }}_{i}+\sigma_{\mu_{i}}{\tilde{W}}_{\mu_{i}}^{T}{\hat{W}}_{\mu_{i}}\right)\\ \; & -\sum_{i=1}^{N}\Psi_{i}\delta \varphi_{i}h\left(W_{f_{i}}^{T}\phi_{f_{i}}+\frac{\delta h\varphi_{i}\theta_{i}\phi_{f_{i}}^{T}\phi_{f_{i}}}{4\gamma_{i}}\right)\\ \; & -\sum_{i=1}^{N}\Psi_{i}\delta \varphi_{i}h\left(\psi_{i}\tanh\left(\frac{\varphi_{i}\Psi_{i}}{\zeta_{i}}\right)+Y_{i}\right)\\ \; & +\sum_{i=1}^{N}\left(-c_{i}\beta_{i}^{2}-h\beta_{i}\mu_{i}\left(u_{i}\left(t-\tau_{i\max}\right)-u_{i}\left(t\right)\right)\right) \end{array}$$

Invoking Young’s inequality, it follows that

(42) $$\sigma_{\theta_{i}}{\tilde{\theta }}_{i}{\hat{\theta }}_{i}\leq -\frac{\sigma_{\theta_{i}}}{2}{{\tilde{\theta }}_{i}}^{2}+\frac{\sigma_{\theta_{i}}}{2}\theta_{i}^{2}$$

(43) $$\sigma_{\psi_{i}}{\tilde{\psi }}_{i}{\hat{\psi }}_{i}\leq -\frac{\sigma_{\psi_{i}}}{2}{{\tilde{\psi }}_{i}}^{2}+\frac{\sigma_{\psi_{i}}}{2}\psi_{i}^{2}$$

(44) $$\sigma_{\mu_{i}}{\tilde{W}}_{\mu_{i}}^{T}{\hat{W}}_{\mu_{i}}\leq -\frac{\sigma_{\mu_{i}}}{2}{{\tilde{W}}_{\mu_{i}}}^{T}{\tilde{W}}_{\mu_{i}}+\frac{\sigma_{\mu_{i}}}{2}W_{\mu_{i}}^{T}W_{\mu_{i}}$$

(45) $$-\Psi_{i}\delta \varphi_{i}hW_{f_{i}}^{T}\phi_{f_{i}}\leq \frac{\delta^{2}\varphi_{i}^{2}h^{2}\Psi_{i}^{2}}{4\gamma_{i}}\theta_{i}\phi_{f_{i}}^{T}\phi_{f_{i}}+\gamma_{i}$$

Based on Assumption 2 and the boundedness of $\mu_{i}$, it can conclude that $\mu_{i}u_{i}\left(t-\tau_{i}\right)-\mu_{i}u_{i}\left(t-\tau_{i\max}\right)$ and $\mu_{i}u_{i}\left(t-\tau_{i\max}\right)-\mu_{i}u_{i}\left(t\right)$ are bounded. This implies that the two positive constants $N_{i1}$ and $N_{i2}$ can be expressed as

(46) $$|\mu_{i}u_{i}\left(t-\tau_{i\max}\right)-\mu_{i}u_{i}\left(t\right)|\leq N_{i1}$$

and

(47) $$|u_{i}\left(t-\tau_{i}\right)-u_{i}\left(t-\tau_{i\max}\right)|\leq N_{i2}$$

From Young’s inequality, we have

(48) $$-\beta_{i}h\mu_{i}\left(u_{i}\left(t-\tau_{i\max}\right)-u_{i}\left(t\right)\right)\leq \frac{1}{2}h\beta_{i}^{2}+\frac{1}{2}hN_{i1}^{2}$$

According to (32), it can be inferred from Assumption 1 that

(49) $$-\Psi_{i}\delta \varphi_{i}hY_{i}\leq \delta h|\Psi_{i}\varphi_{i}Y_{i}|\leq \delta h\varphi_{i}\left|\Psi_{i}\psi_{i}\right|$$

where $\psi_{i}=\varpi_{i\max}+\varepsilon_{f_{i\max}}+\varepsilon_{\mu_{i\max}}+N_{i2}$.

Incorporating (42)–(49) into (41), we can obtain

(50) $$\begin{array}{cc} \dot{V} & \leq -\sum_{i=1}^{N}\left(k_{i}\Psi_{i}^{2}+\frac{\sigma_{\theta_{i}}}{2}{\tilde{\theta }}_{i}^{2}+\frac{\sigma_{\psi_{i}}}{2}{\tilde{\psi }}_{i}^{2}+\frac{\sigma_{\mu_{i}}}{2}{\tilde{W}}_{\mu_{i}}^{T}{\tilde{W}}_{\mu_{i}}\right)\\ \; & \;+\sum_{i=1}^{N}\delta h\psi_{i}\left(\left|\Psi_{i}\varphi_{i}\right|-\Psi_{i}\varphi_{i}\tanh\left(\frac{\Psi_{i}\varphi_{i}}{\zeta_{i}}\right)\right)\\ \; & \;+\sum_{i=1}^{N}\left(\frac{\sigma_{\theta_{i}}}{2}\theta_{i}^{2}+\frac{\sigma_{\psi_{i}}}{2}\psi_{i}^{2}+\frac{\sigma_{\mu_{i}}}{2}W_{\mu_{i}}^{T}W_{\mu_{i}}+\gamma_{i}\right)\\ \; & \;-\sum_{i=1}^{N}\left(c_{i}-\frac{h}{2}\right)\beta_{i}^{2}+\sum_{i=1}^{N}\frac{1}{2}hN_{i1}^{2} \end{array}$$

Based on Lemma 1, we have

(51) $$|\varphi_{i}\Psi_{i}|-\varphi_{i}\Psi_{i}\tanh\left(\frac{\varphi_{i}\Psi_{i}}{\zeta_{i}}\right)\leq 0.2785\zeta_{i}$$

Combining (50) and (51), we have

(52) $$\begin{array}{cc} \dot{V}\leq & -\sum_{i=1}^{N}\left(k_{i}\Psi_{i}^{2}+\frac{\sigma_{\theta_{i}}}{2}{\tilde{\theta }}_{i}^{2}+\frac{\sigma_{\psi_{i}}}{2}{\tilde{\psi }}_{i}^{2}+\frac{\sigma_{n_{i}}}{2}{\tilde{W}}_{\mu_{i}}^{T}{\tilde{W}}_{\mu_{i}}\right)\\ \; & -\sum_{i=1}^{N}\left(c_{i}-\frac{h}{2}\right)\beta_{i}^{2}+\sum_{i=1}^{N}0.2785\delta h\psi_{i}\zeta_{i}\\ \; & +\sum_{i=1}^{N}\left(\frac{\sigma_{\theta_{i}}}{2}\theta_{i}^{2}+\frac{\sigma_{\psi_{i}}}{2}\psi_{i}^{2}+\frac{\sigma_{n_{i}}}{2}W_{\mu_{i}}^{T}W_{\mu_{i}}+\frac{h}{2}N_{i1}^{2}+\gamma_{i}\right) \end{array}$$

Then, rewrite (52) as

(53) $$\dot{V}\leq -\xi V+\eta$$

where

(54) $$\xi =\min_{1\leq i\leq N}\left\{2k_{i},F_{\theta_{i}}\sigma_{\theta_{i}},F_{\psi_{i}}\sigma_{\psi_{i}},F_{\mu_{i}}\sigma_{\mu_{i}},2c_{i}-h\right\}$$

(55) $$\begin{array}{cc} \eta = & \sum_{i=1}^{N}\left(\gamma_{i}+\frac{\sigma_{\theta_{i}}}{2}\theta_{i}^{2}+\frac{\sigma_{\psi_{i}}}{2}\psi_{i}^{2}+\frac{\sigma_{\mu_{i}}}{2}W_{\mu_{i}}^{T}W_{\mu_{i}}+0.2785\delta \Psi_{i}h\psi_{i}\zeta_{i}+\frac{h}{2}N_{i1}^{2}\right) \end{array}$$

Take (53), multiply its each sides by $e^{\xi t}$, and integrate over the range [0,t], we can obtain

(56) $$\int_{0}^{t}\frac{d(V\left(t\right)e^{\xi t})}{dt}dt\leq \int_{0}^{t}\eta e^{\xi t}dt$$

Then, (56) can be described as

(57) $$V\leq \frac{\eta }{\xi }+\left(V\left(0\right)-\frac{\eta }{\xi }\right)e^{-\xi t}$$

From (57), we have $lim_{t\to +\infty }$V(t) = η/ξ. This means that $\Psi_{i}$, $\beta_{i}$, ${\tilde{\theta }}_{i}$, ${\tilde{\psi }}_{i}$ and ${\tilde{W}}_{\mu_{i}}$ are ultimately bounded in a small neighborhood of zero. Based on Assumption 1, it is proven that $x_{i}$, $v_{i}$, and $a_{i}$ are also bounded, indicating that the individual stability can be achieved.

#### 3.3.2. String Stability

String stability analysis is established as follows [42]. As defined in (25), when $\Psi_{i}$ converges to a small neighborhood around the origin by selecting appropriate design parameters, it can be derived that

(58) $$\delta s_{i}\left(t\right)-s_{i+1}\left(t\right)=0$$

Since $s_{i}\left(t\right)={\dot{\psi }}_{i}+\lambda \psi_{i}$, one has

(59) $$\delta \left({\dot{\psi }}_{i}+\lambda \psi_{i}\right)={\dot{\psi }}_{i+1}+\lambda \psi_{i+1}$$

Take the Laplace transform of both sides of (59), we have

(60) $$\delta \left(sE_{i}\left(s\right)+\lambda E_{i}\left(s\right)\right)=sE_{i+1}\left(s\right)+\lambda E_{i+1}\left(s\right)$$

where $E_{i}\left(s\right)$ is the Laplace transform of $\psi_{i}$.

According to (60), the transfer function of the transformed error is

(61) $$G_{i}\left(s\right)=\frac{E_{i+1}\left(s\right)}{E_{i}\left(s\right)}=\delta$$

According to (20), $\psi_{i}$ has the same monotonicity as the $e_{i}^{m}$. Assuming $E_{i}^{m}\left(s\right)$ denotes the Laplace transform corresponding to $e_{i}^{m}\left(t\right)$, the transfer function of the spacing error is

(62) $$G_{i}^{m}\left(s\right)=\frac{E_{i+1}^{m}\left(s\right)}{E_{i}^{m}\left(s\right)}=\delta$$

Substituting the design parameter 0<δ≤1, we have $|G_{i}^{m}\left(jw\right)|\leq 1$. Furthermore, the $H_{\infty }$ norm is computed as $||\;G_{i}^{m}\left(s\right){\left|\right|}_{\infty }\leq 1$. Therefore, the string stability of the platoon is ensured when 0<δ≤1, and the proof is completed [43].

## 4. Numerical Examples

In this part, the numerical examples are presented to verify the effectiveness and superiority of the proposed method.

A platoon, including one leader vehicle and four followers, is adopted in this work. The parameters of the vehicle platoon (3) are $\varsigma_{i}\left(t\right)=0.5m_{i}\left[1+0.5sin\left(tx_{i}\right)\right]$, $m_{i}=1500\;\mathrm{kg}$, $A_{i}=2\;m^{2}$, $\kappa_{i}=0.5$, $m_{ai}=1.2\;{\mathrm{kg}/m}^{3}$, $F_{mi}=5\;N$, $d_{mi}=5$, $\tau_{i}=0.15-0.1\sin\left(t\right)$, $C_{di}=0.35$, and $\varpi_{i}=0.1\cos\left(t\right)$. The parameters of (14) are chosen as $\Delta_{i}=7\;m$, $l_{i}=4\;m$, $K_{i}=0.8$, and h=0.14s. The prescribed performance function ρ=(0.1−0.02)exp(−0.3t)+0.02, and $\ell_{\min}=\ell_{\max}=1$. The vehicle platoon’s initial states are shown in Table 1.

Define the leader’s acceleration as [24]

(63) $$\begin{array}{cc} a_{0}\left(t\right) & =\left\{\begin{array}{cc} 0.5\;{m/s}^{2}, & 0\leq t<4\;s,\\ 2\;{m/s}^{2}, & 4\leq t<8\;s,\\ -0.5t+6\;{m/s}^{2}, & 8\leq t<12\;s,\\ 0\;{m/s}^{2}, & 12\leq t<16\;s,\\ -2\;{m/s}^{2}, & 16\leq t<20\;s,\\ 0\;{m/s}^{2}, & 20\leq t<23\;s,\\ 3\;{m/s}^{2}, & 23\leq t<26\;s,\\ 0\;{m/s}^{2}, & \mathrm{otherwise} \end{array}\right. \end{array}$$

### 4.1. Results of the Proposed Control Method

To verify the effectiveness of the proposed method, numerical examples are carried out by employing the developed controller (33) and adaptive updating mechanisms (34)–(36) under the topology structure illustrated in Figure 1. In the RBFNN approximation, the uniform grid method is adopted to determine the center points and widths of RBFNN. The velocity and acceleration are confined within a compact working domain, and the state variables are uniformly discretized within this region. Nine neural nodes are used with the center of input vector $Z_{i}={\left[v_{i},a_{i}\right]}^{T}$ evenly spaced in $\left[-15\;m/s,15\;m/s\right]\times \left[-5\;{m/s}^{2},5\;{m/s}^{2}\right]$, and the basis function width is set to $b_{k}=2$. Control parameters are listed as: $\beta_{i}\left(0\right)=0$, $\theta_{i}\left(0\right)=0$, Γ=5, $k_{i}=0.5$, δ=0.95, $\gamma_{i}=0.5$, $\zeta_{i}=10$, λ=140, $F_{\theta_{i}}=15$, $F_{\psi_{i}}=2$, $F_{\mu_{i}}=0.5$, $\sigma_{\theta_{i}}=0.01$, $\sigma_{\psi_{i}}=0.001$, $\sigma_{\mu_{i}}=0.01$, $c_{i}=1.5$. $T_{1}=8\;s$, $T_{2}=10\;s$, $T_{3}=12\;s$, $T_{4}=14\;s$.

**Figure 2:**
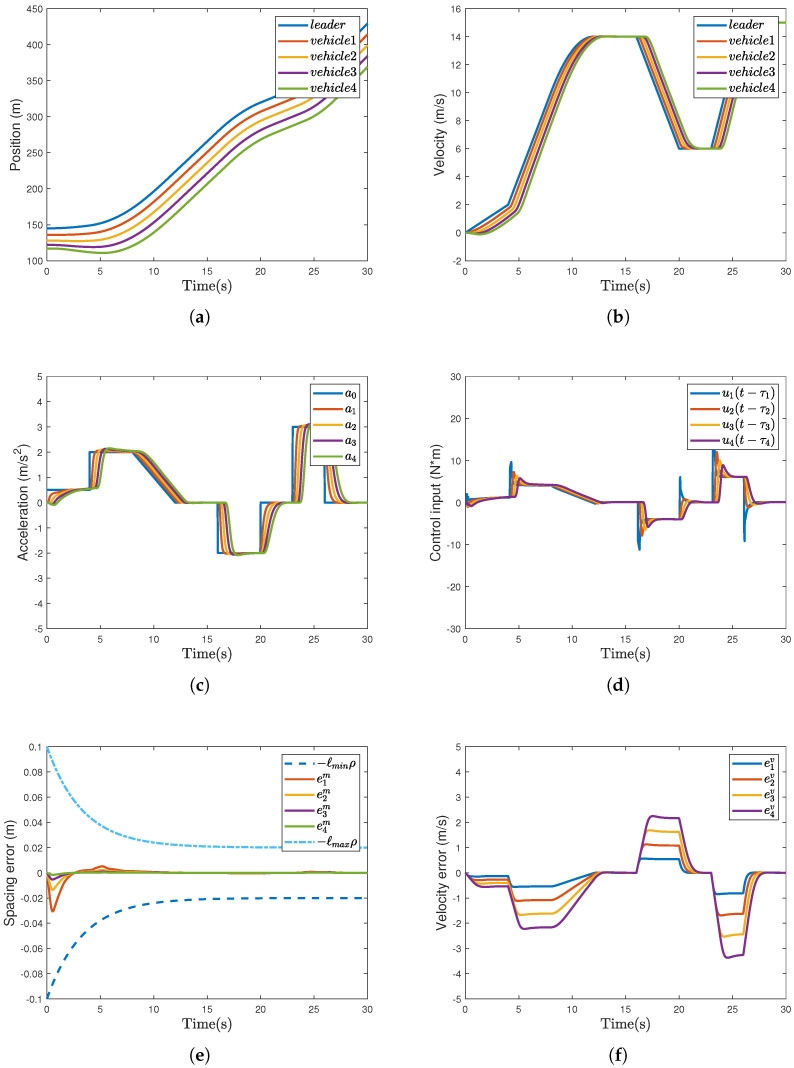
The results of the controller incorporating the improved ICM and the RBFNN−AUM (36). (**a**) Position $x_{i}$ (m). (**b**) Velocity $v_{i}$ (m/s). (**c**) Acceleration $a_{i}$ ($m/s^{2}$). (**d**) Control input $u_{i}\left(t-\tau_{i}\right)$ (N*m). (**e**) Spacing error $e_{i}^{m}$ (m). (**f**) Velocity error $e_{i}^{v}$ (m/s).

The example results are illustrated in Figure 2. The position curves are provided in Figure 2a. The lack of crossing or overlapping among vehicle paths demonstrates that the proposed control method effectively avoids the occurrence of inter-vehicle collisions. Figure 2b shows that velocity synchronization is achieved in the platoon. As illustrated in Figure 2c, the accelerations of all follower vehicles rapidly converge to the leader’s acceleration. Figure 2d shows the control input profiles with time delays. It can be observed from Figure 2e that $e_{i}^{m}$ converges to a prescribed region in predefined time, indicating that the proposed strategy achieves the prescribed tracking performance. As shown in Figure 2f, the proposed strategy enables $e_{i}^{v}$ to rapidly converge to a small neighborhood of zero.

To provide a quantitative analysis of the theoretical stability results derived from the Lyapunov inequality $\dot{V}\leq -\xi V+\eta$, we computed the values of ξ=0.002 and η=2.805 using the control gains selected for the numerical examples. Combining $lim_{t\to +\infty }$V(t) = η/ξ=2.805/0.002=1402.5, it can be derived that $\Psi_{i}^{2}\leq 2V=2805$, which means $|\Psi_{i}|\leq \sqrt{2805}=52.96$. According to the error mapping transformation, the ultimate bound of the spacing error is $|e_{i}^{m}|\leq 52.96\rho_{\max}\left(t\right)/\lambda =0.0398$ m. It is inferred from (54) and (57) that an increase of $k_{i}$ is beneficial for improving the transient response performance and steady-state control accuracy, yet it will result in higher control energy consumption. Therefore, a trade-off between control performance and cost is usually required when selecting design parameters.

### 4.2. Results of the Comparative Numerical Example

To validate the superiority of the proposed strategy, comparative numerical examples are presented based on the ICM proposed in [24]. All comparisons are conducted under identical initial conditions, leader acceleration, external disturbances, and sampling settings to ensure fairness. The difference is that the parameters are chosen as $K_{i}=1$ and $\mu_{i}\left(t\right)=1$. Although the control method in [24] achieves the prescribed performance, the design of constant parameters limits the platoon’s generality and robustness. Table 2 provides a quantitative comparison between the two methods in terms of root−mean−square errors (RMSE), maximum overshoot, and settling time of $e_{i}^{m}$.

The RMSE of the spacing error can be obtained by calculating the following equation

(64) $$RMSE=\frac{1}{4}\sum_{i=1}^{4}\sqrt{\frac{1}{N}\sum_{k=1}^{N}{\left(e_{i}^{m}\right)}^{2}\left(k\right)}$$

where *N* denotes the total number of time steps.

The maximum overshoot $M_{p}$ of the spacing error is defined as

(65) $$M_{p}=\max_{t}\left|e_{i}^{m}\left(t\right)\right|\times 100\%$$

The settling time is defined as the time instant when the spacing error enters and remains within a range of ±0.005 m, as follows

(66) $$t_{s,i}=\min\left\{t||e_{i}^{m}\left(t_{a}\right)|\leq \epsilon ,\forall t_{a}\geq t\right\}$$

where $t_{a}$ is an auxiliary time variable, and ϵ=0.005 denotes the error tolerance.

**Figure 3:**
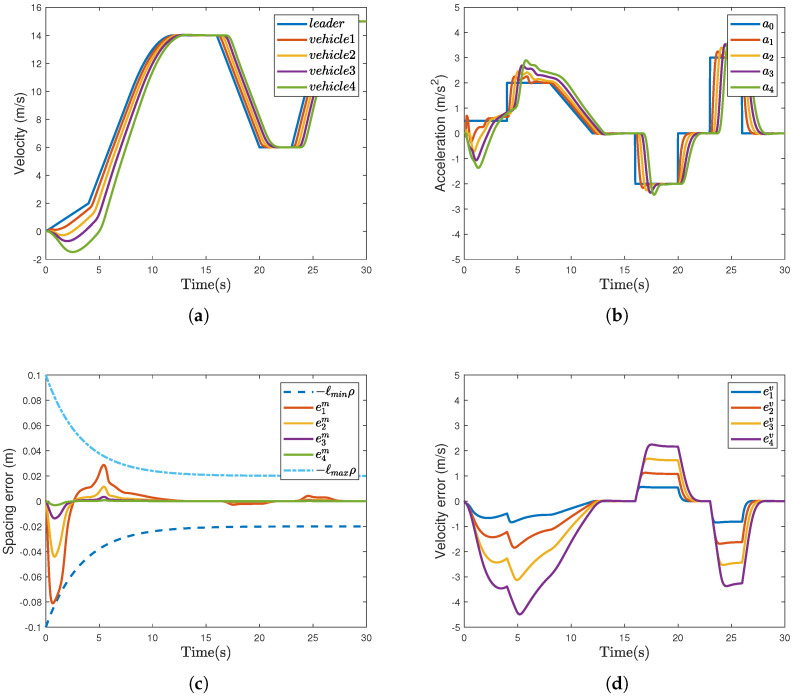
The results of the controller based on the ICM proposed in [24]. (**a**) Velocity $v_{i}$ (m/s). (**b**) Acceleration $a_{i}$ ($m/s^{2}$). (**c**) Spacing error $e_{i}^{m}$ (m). (**d**) Velocity error $e_{i}^{v}$ (m/s).

Figure 3a–d show the profiles of vehicles’ velocity, acceleration, spacing error, and velocity error, respectively. As shown in Figure 2b and Figure 3a, it can be observed that the proposed strategy achieves smaller velocity fluctuations and faster response speed. Referring to Figure 3b, it is found that the acceleration profiles in Figure 2c are smoother. Moreover, according to Figure 3c and Table 2, we can observe that the proposed method improves the vehicle’s tracking accuracy by 70.8%, reduces the maximum overshoot of $e_{i}^{m}$ by 62.2%, and decreases the settling time by 39.7%. In light of Figure 3d, we can observe that $e_{i}^{v}$ has a smaller peak in Figure 2f, indicating that the proposed strategy achieves superior transient performance.

To further validate the robustness and generality of the proposed method, a numerical example is presented under the leader−follower (LF) topology, with all parameters identical to those in the proposed method. The quantitative performance comparison of the proposed method under the LF topology is presented in Table 3.

**Figure 4:**
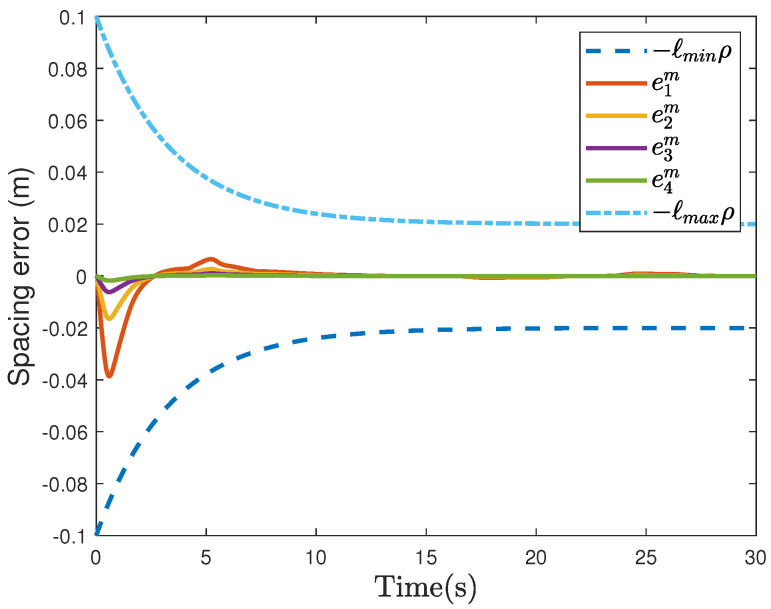
The results of the controller incorporating the improved ICM and RBFNN−AUM under the LF topology.

Based on Figure 4 and Table 3, the proposed method demonstrates superior performance under the LF topology compared to the approach in [24]. Specifically, the vehicle’s tracking accuracy is improved by 63.9%, the maximum overshoot is reduced by 52.4%, and settling time is decreased by 35.6%. The above results fully demonstrate that even under the leader-follower topology, the proposed control method is still significantly superior to the comparative methods in terms of all key performance metrics, verifying its robustness and generality.

Additionally, to evaluate the controller’s robustness under uncertain input delays and disturbances, we carried out 50 independent numerical examples. In each example, the input delays $\tau_{i}$ are randomly selected within the range of [0,0.25] s, and the disturbances $\varpi_{i}$ are randomly chosen within [−0.1,0.1]. The resulting spacing error RMSE values are shown in Table 4. The data show that the proposed control method can maintain a small RMSE of spacing error under different input delays $\tau_{i}$ and disturbances $\varpi_{i}$. Moreover, even when the input delays $\tau_{i}$ and disturbances $\varpi_{i}$ reach their maximum values, the platoon can still achieve the desired control performance, which fully verifies the excellent robustness of the proposed method. As demonstrated in the previous analysis, the proposed method yields better performance compared to existing approach.

## 5. Conclusions

This study investigates the adaptive SMC problem for vehicle platoon with input delays and unknown time-varying control coefficients. To enhance the robustness of the vehicle platoon, an improved ICM incorporating the adjustment factor is proposed to deal with the detrimental effects of input delays. To effectively handle the negative impact of unknown time-varying control coefficients on the platoon performance, a novel RBFNN−AUM is proposed to enhance the platoon’s tracking performance. By employing the improved ICM and the RBFNN−AUM, a new adaptive SMC strategy is presented with the aim of achieving the platoon’s control objectives. The comparative numerical examples confirm the superiority and effectiveness of the proposed method. It is worth noting that the effectiveness of control method proposed relies on the fundamental Assumption 2 that the input delays are bounded (i.e., $\tau_{i}\left(t\right)\leq \tau_{i\max}$) [24,44,45]. Future work will focus on investigating deep reinforcement learning-based control issues of the vehicle platoon subject to communication failure.

## Figures and Tables

**Table 1:** The initial states of the vehicle platoon.

i	0	1	2	3	4
$x_{i}\left(0\right)$	145.0	136.0	128.0	122.0	117.0
$v_{i}\left(0\right)$	0.0	0.0	0.0	0.0	0.0
$a_{i}\left(0\right)$	0.5	0.0	0.0	0.0	0.0

**Table 2:** Performance comparison under different Methods.

Performance Metric	The ICM [24]	The Improved ICM and the RBFNN−AUM (36)
RMSE	0.0072	0.0021
$M_{p}$	0.0810	0.0306
$t_{s,i}$	8.670	5.228

**Table 3:** Performance comparison under different platoon topologies.

Performance Metric	The ICM [24]	The Improved ICM and the RBFNN−AUM (36) Under the LF Topology
RMSE	0.0072	0.0026
$M_{p}$	0.0810	0.0386
$t_{s,i}$	8.670	5.590

**Table 4:** The Example results under different input delays $\tau_{i}$ and disturbance amplitudes $\varpi_{i}$.

$\tau_{i}$	$\varpi_{i}$	RMSE	$\tau_{i}$	$\varpi_{i}$	RMSE
0.080	−0.042	0.0019	0.097	−0.055	0.0018
0.164	−0.056	0.0026	0.127	0.019	0.0023
0.152	0.014	0.0034	0.100	−0.015	0.0026
0.117	0.032	0.0029	0.099	−0.048	0.0019
0.101	−0.052	0.0020	0.100	−0.060	0.0020
0.102	−0.073	0.0027	0.099	−0.073	0.0030
0.151	−0.013	0.0029	0.104	−0.010	0.0023
0.124	0.029	0.0023	0.158	0.025	0.0031
0.250	0.100	0.0042	0.127	−0.018	0.0022
0.157	0.011	0.0028	0.175	0.023	0.0028
0.121	−0.033	0.0030	0.177	−0.013	0.0028
0.157	0.008	0.0032	0.108	0.032	0.0020
0.067	−0.007	0.0023	0.155	0.020	0.0025
0.153	−0.010	0.0035	0.130	0.037	0.0021
0.096	−0.019	0.0019	0.148	0.030	0.0031
0.143	0.049	0.0027	0.200	0.033	0.0039
0.148	−0.036	0.0031	0.124	0.011	0.0025
0.114	−0.023	0.0026	0.234	−0.067	0.0036
0.105	0.019	0.0022	0.085	−0.032	0.0018
0.140	0.005	0.0029	0.119	−0.013	0.0026
0.193	−0.054	0.0039	0.090	0.021	0.0020
0.116	0.006	0.0027	0.127	−0.047	0.0028
0.034	−0.009	0.0015	0.137	0.013	0.0026
0.177	−0.015	0.0028	0.198	0.007	0.0033
0.176	−0.014	0.0037	0.060	−0.052	0.0021

## Data Availability

The original contributions presented in the study are included in the article; further inquiries can be directed to the corresponding author.

## Abbreviations

The following abbreviations are used in this manuscript:

SMC	Sliding Mode Control
ICM	Integral Compensation Mechanism
RBFNN	Radial Basis Function Neural Network
RBFNN−AUM	RBFNN-based adaptive updating mechanism
RMSE	Root−Mean−Square Errors
LF	Leader−Follower
